# The Distributional Ecology of the Maned Sloth: Environmental Influences on Its Distribution and Gaps in Knowledge

**DOI:** 10.1371/journal.pone.0110929

**Published:** 2014-10-22

**Authors:** Danielle de Oliveira Moreira, Gustavo Rocha Leite, Marinez Ferreira de Siqueira, Bruno Rocha Coutinho, Mariana Santos Zanon, Sérgio Lucena Mendes

**Affiliations:** 1 Programa de Pós-graduação em Ciências Biológicas (Biologia Animal), Departamento de Ciências Biológicas, Universidade Federal do Espírito Santo, Vitória, Espírito Santo, Brazil; 2 Unidade de Medicina Tropical, Departamento de Patologia, Universidade Federal do Espírito Santo, Vitória, Espírito Santo, Brazil; 3 Instituto de Pesquisas do Jardim Botânico do Rio de Janeiro, Rio de Janeiro, Brazil; 4 Secretaria de Extrativismo e Desenvolvimento Rural Sustentável, Ministério do Meio Ambiente, Brasília, Brazil; University of Western Ontario, Canada

## Abstract

The maned sloth *Bradypus torquatus* (Pilosa, Bradypodidae) is endemic to a small area in the Atlantic Forest of coastal Brazil. It has been listed as a threatened species because of its restricted geographic range, habitat loss and fragmentation, and declining populations. The major objectives of this study were to estimate its potential geographic distribution, the climatic conditions across its distributional range, and to identify suitable areas and potential species strongholds. We developed a model of habitat suitability for the maned sloth using two methods, Maxent and Mahalanobis Distance, based on 42 occurrence points. We evaluated environmental variable importance and the predictive ability of the generated distribution models. Our results suggest that the species distribution could be strongly influenced by environmental factors, mainly temperature seasonality. The modeled distribution of the maned sloth included known areas of occurrence in the Atlantic Forest (Sergipe, Bahia, Espírito Santo, and Rio de Janeiro), but did not match the observed distributional gaps in northern Rio de Janeiro, northern Espírito Santo or southern Bahia. Rather, the model showed that these areas are climatically suitable for the maned sloth, and thus suggests that factors other than climate might be responsible for the absence of species. Suitable areas for maned sloth were located mainly in the mountainous region of central Rio de Janeiro throughout Espírito Santo and to the coastal region of southern Bahia. We indicate 17 stronghold areas and recommended survey areas for the maned sloth. In addition, we highlight specific areas for conservation, including the current network protected areas. Our results can be applied for novel surveys and discovery of unknown populations, and help the selection of priority areas for management and conservation planning, especially of rare and relatively cryptic species directed associated with forested habitats.

## Introduction

How to conserve a species in its geographic range, especially rare and threatened species, is the great question of Conservation Biology. Concerns about biodiversity arise because present extinction rates are exceptionally high [1], and many threatened species are geographically concentrated in some regions [2], especially in the hotspots of biodiversity [2], [3]. Considering that conservation biologists are often pushed to make recommendations about conserving biodiversity despite limited species-distribution data [4]–[7], it is extremely important to understand how a species is distributed geographically and which habitats are environmentally suitable.

Useful niche-based approaches that identifies regions containing suitable environmental conditions based on habitat characteristics at locations of known species occurrences has been extensively applied in the last 20 years [8], [9]. They are correlative species distribution modeling (SDM) [8] and these methods are required for many studies, for example, to predict ecological niche and geographical range of species [10]–[13], to create maps for conservation planning, and to manage species of conservation concern by predicting the occurrence of rare and threatened species [14]–[17]. When the geographic distribution of a species is poorly documented or have gaps of information, SDM can be used to predict possible and presumed occupied locations, and so potentially inform prioritizations efforts [18]. In this paper, we present an ecological niche model for predicting suitable habitat for the maned sloth (*Bradypus torquatus*) within the Brazilian Atlantic Forest.

The maned sloth (*Bradypus torquatus* Illiger, 1811) is an endemic mammal to the Atlantic Forest of northeastern and southeastern Brazil, from the state of Sergipe to the state of Rio de Janeiro [19]–[21]. Following the pygmy sloth (*Bradypus pygmaeus*), the maned sloth is the most threatened *Bradypus* species [22]. It is assessed as Vulnerable in the IUCN Red List of Threatened Species owing to its restricted geographic range, habitat loss and fragmentation, and declining populations [23]. Its estimated area of occupancy, based on remaining forest within its range, is less than 1,000 km^2^ [23], which increases the importance of new studies that support conservation planning and prioritization in reserve selection.

Our objectives in this study were (1) to develop a potential geographic distributional model for *B*. *torquatus*, considering its tolerance to topographic and climatic conditions; (2) to identify suitable climatic conditions in unknown regions, mainly in those discontinuous (gap) areas; (3) to estimate the total area of forest fragments within its most suitable areas; and (4) indicate species strongholds for conservation purposes.

## Materials and Methods

### Identity, ecology and distribution of *Bradypus torquatus*

The genus *Bradypus* (Pilosa, Bradypodidae) consists of four species of three-toed sloths distributed throughout the Neotropical region [24]. The pygmy sloth (*Bradypus pygmaeus* Anderson & Handley, 2001) is restricted to the small Isla Escudo de Veraguas Island off the Caribbean coast of Panama, and it is the smallest species of three-toed sloths [24]. The pale-throated sloth (*Bradypus tridactylus* Linnaeus, 1758) occurs between the Orinoco River and the Amazon River in northern South America. The brown-throated sloth (*Bradypus variegatus* Shinz, 1825) has a wider geographic distribution ranging from southern Central America to South America [19]–[21]. The maned sloth is sympatric with the brown-throated sloth in some areas [19]–[21].

The maned sloth is characterized by a mane of black hairs projecting over the shoulders [25]. It occurs from sea level to 1,290 m [21], most typically in areas with annual precipitation of 1,200 mm or higher. Sloths are a nonhibernating animal with relatively low metabolic rates and low body temperature compared to other mammals. They are poor regulators of body temperature with little ability to increase their metabolism due to their small muscle mass [26]. Thus, they are intolerant to large variations in temperature and are susceptible to cold conditions.

Regarding the geographic distribution, experts debate whether the historical occurrence of maned sloth extended beyond São Francisco River, toward the north, reaching the state of Pernambuco or, even, the state of Rio Grande do Norte [27]. Nonetheless, according to Hirsh and Chiarello [21], that was believed to be unlikely. Also, the known populations of maned sloth are in restricted and discontinuous regions in the states of Bahia, Espírito Santo, and Rio de Janeiro, separated by apparent distributional gaps between Mucuri River and Doce River in southern Bahia and northern Espírito Santo, and other one between southern Espírito Santo and northern Rio de Janeiro [20], [21], [28]. However, there are still a lot of unconfirmed records and gaps of knowledge about the species’ geographic range, mainly because it is a difficult animal to see in the wild due its behavior [29], its cryptic nature [22], [30] and misidentification.

### Study area

**Figure 1:**
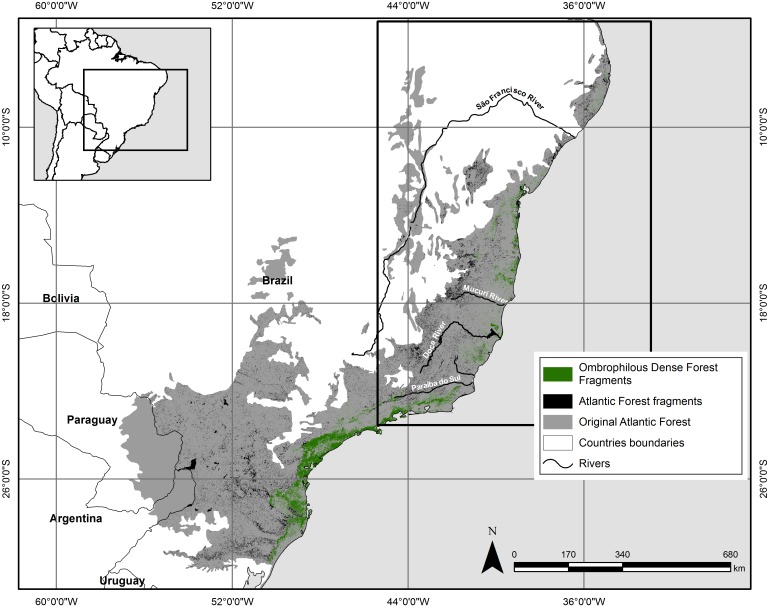
Original and current extent of the Atlantic Forest region in Brazil. The black box corresponds to the extent of the maned sloth’s extent. Cartographic bases: [32], [57], [58], [79]. Geographic Projection: Datum WGS 1984.

The Brazilian Atlantic Forest originally stretched from the state of Rio Grande do Norte, in northeastern Brazil, to Rio Grande do Sul, the southernmost Brazilian state (Figure 1). In northeastern Brazil, it occupies a thin coastal strip at low elevations, while in the south the Atlantic Forest extends far inland along the slopes of the Serra do Mar to high elevations in southern Minas Gerais, Rio de Janeiro, and the state of São Paulo [31]. The original forest cover has been reduced by approximately 90% in some areas (Figure 1) [32], and it hosts about 70% of Brazil’s threatened animal species [33].The Atlantic Forest is one of the top five biodiversity hotspots in the world, and is considered the highest conservation priority in Brazil because of the high level of habitat destruction and fragmentation [34].

### Presence data

We compiled 84 occurrence records of the maned sloth from the scientific literature and museum collections, excluding interview and captivity data. Some authors argue that museum records are not reliable sources of locations to be used in SDMs because of spatial errors caused by of a variety of factors such as localities that can be described only as named places to poor precision of the geographic coordinates [35]. But when appropriate precautions are taken, they showed to be important, especially when there are small sample sizes available [36].

Some studies have demonstrated the great potential of using museum records for biodiversity studies, revealing that even using imprecise data, predictive models can be still fairly robust to locational error (e.g. Loiselle et al. [4]; Graham et al. [35]; Newbold [7]). Taking into consideration that the objective of our data was for habitat suitability modeling, and not for obtaining an exhaustive list of occurrence records, and that we preferred to be conservative, discarding any records with incomplete, inaccurate or dubious information, we used only 46 localities of occurrence to model the species distribution. We georeferenced all reliable records using the centroid of the site boundaries in question (i.e. reserves, towns or counties). If the boundary area of the locality where the species was recorded was larger than the pixel size used in the analysis (approximately 4.5 km), we did not use this record in modeling. We also discarded redundant points, i.e., when more than one point fell into the same grid pixel. This process resulted in 42 presence points (see Table S1) that were projected in a Geographic Information System environment (GIS) using the World Geographic System 1984 (WGS 1984).

### Environmental variables

We obtained 19 bioclimatic data layers and the digital elevation model from the WorldClim database [37] for the Atlantic Forest region. All variables were raster datasets, with a resolution of 0.042° (approximately 4.5 km). The bioclimatic layers are based on compiled averages of climate as measured at weather stations from a large number of sources from 1950–2000 period, which were spatially interpolated on grids [37]. The elevation model was based on elevation data obtained by the Shuttle Radar Topography Mission (SRTM) that generated a complete high-resolution digital topographic database of Earth in the year 2000.

For selection of nonredundant environmental variables for the modeling, we calculated Pearson correlation coefficients for all combinations of the environmental layers and removed single layers that were highly correlated pairwise (|r|≥0.80). Variables representing extreme environmental conditions were preferably maintained because they have significant effects on species [38], [39]. The remaining nine environmental layers used for the modeling were: elevation, mean monthly temperature range, temperature seasonality, maximum temperature of warmest month, minimum temperature of coldest month, temperature annual range, annual precipitation, precipitation of wettest month, and precipitation of driest month.

### Modeling approach

We used two modeling approaches in this study: Maximum Entropy (Maxent) and Mahalanobis Distance.

Maxent (version 3.3.1; http://www.cs.princeton.edu/~schapire/maxent/) is a general-purpose machine learning approach, which uses a mathematical formulation for modeling species’ potential geographic distributions with presence-only data [40], [41]. Previous presence-only distribution modeling attempts suggest Maxent performs better than other algorithms (e.g. GARP, DOMAIN, and BIOCLIM) [40], [42]. We used the default settings of Maxent.

Mahalanobis Distance is a generic algorithm based on environmental dissimilarity metrics. This metric is an extension of the standardized Euclidian Distance by taking into account the covariance structure amongst the predictor variables [43]. Mahalanobis Distance is more complex because it considers the covariance matrix among environmental variables in the occurrence points. Therefore, we can interpret the model as an expression of environmental restrictions on species, including correlations among variables [44]. This algorithm ranks potential sites by their Mahalanobis Distance to a vector expressing the mean environmental conditions (i.e. the centroid) of all the occurrence records in environmental space [45]. Although this algorithm has not been well explored in species distribution modeling, simulations by Allouche et al. [45] showed a steep increase in predictive accuracy with increasing sample size reaching asymptotic values at samples sizes of 20–50 records. The model was implemented within MATLAB environment (The MathWorks, Inc., Natick, MA, USA).

In both algorithms, we used the bootstrapping method (10 partitions) to divide our 42 presence points into 70% training data (to generate the model), and 30% testing data (to test the model). We ran both algorithms on each of the 10 partitions, for a total of 20 model iterations.

### Model evaluation

We evaluated the predictive ability of the generated potential geographic distribution models with two methods. First, we used the receiver operating characteristic (ROC) plot method. An ROC plot is created by plotting the sensitivity values (the true-positive fraction) against 1 – specificity (the false-positive fraction) for all available probability thresholds [46]. The AUC derived from the ROC plot can be interpreted as a measure of the ability of the algorithm to discriminate between a suitable environmental condition and a random analysis pixel [40]. To minimize problems with this measure, we followed some recommendations of Lobo et al. [47] and Peterson et al. [48] such as constraining our analysis to the original extent of Atlantic Forest, and averaging the AUC from 10 replicates of the original presence data. A ‘10th percentile training presence’ ‘minimum training presence’ (explanation below) was selected as the threshold value.

Another method of model evaluation was a threshold dependent validation where we evaluated the performance of presence-absence by the True Skill Statistics (TSS), given by: TSS = sensitivity+specificity–1 [49]; where sensitivity is the proportion of correctly predicted presences to their total number and specificity is the proportion of correctly predicted absent cells to the total number, both in the validation dataset. TSS ranges from −1 to +1, where +1 indicates perfect agreement and values of zero or less indicate a performance no better than random. We followed Jones et al. [50] considering values higher than 0.6 good, between 0.2 to 0.6 fair to moderate, and lower than 0.2 poor. We created 12,000 background points (100 to each point of presence test * 10 partitions), following Lobo and Tognelli [51], and we calculated the TSS value for each partition. Alouche et al. [49] recommend the TSS as a simple and intuitive measure for the performance of SDMs when predictions are expressed as presence–absence maps.

### Potential geographic distribution and habitat suitability

The modeling algorithms produce raster pixels in which each cell has a value that summarizes habitat suitability conditions for the species. But, transforming the results of species distribution modeling from probabilities of species occurrence to presence/absence needs a specific threshold [52]. We chose the ‘10th percentile training presence’ value for each of the 10 partitions resulting from the Maxent and Mahalanobis Distance algorithms. The ‘10th percentile training presence’ threshold predicts absent the 10% most extreme presence observations, as these may represent recording errors, ephemeral populations, migrants, or the presence of unusual microclimatic conditions within a cell [53]. The ‘10th percentile training presence’ threshold can indicate the most suitable areas for the maned sloth, increasing, in that way, the possibility of success. Also, because maned sloth has lost most of its habitat due to forest fragmentation, indicating a suitability area with possible occurrence of the species might be interesting for conservation management, including reintroduction programs for the species.

The thresholding procedure converts the raster pixels values of the continuous prediction to binary values, 0 and 1, where 0 means not predicted and 1 means predicted. The raster pixels identified as predicted can be interpreted being at least as suitable as those where the species has been recorded in nature [54]. Applying this thresholding rule, raster pixels with values equal to or higher than the 10th percentile training presence value are considered suitable, and pixels with values below it are considered unsuitable [55]. We then averaged the 10 partitions from both algorithms separately to create potential geographic distribution maps from each algorithm. Finally, we combined and averaged both Maxent and Mahalanobis Distance models to create a single binary ensemble map of potential geographic distribution.

We created a second ensemble map with continuous values to show the highest and lowest suitable values (ranging from 0 to 1). To do so, we retrieved the continuous pixel values from each of the 10 partitions of the Maxent and Mahalanobis Distance models. We multiplied each model replicate by the corresponding thresholded (binary) map separately. Pixels values with a “1” in the binary model remained a continuous value, and pixels with a “0”, remained zero. So in essence, we maintained the continuous values above the threshold value while converting all values below the threshold to zero, thus only those values in the top 10% were kept. This procedure allowed retrieving the continuous values, while maintaining the threshold information. This was done for each of the 10 models per algorithm and these were subsequently averaged. The final Maxent and Mahalanobis Distance models were averaged again to create a continuous ensemble map. It was possible to distinguish the maned sloth habitat suitability by identifying areas of unsuitable (0 to <0.098), low (0.098 to 0.286), medium low (0.286 to 0.462), medium high (0.462 to 0.658), and high suitability (0.658 to 1). We used the ArcGIS natural breaks (Jenks) to define those values for the entire suitable maned sloth habitat.

In sum, we created two ensemble maps, one categorical (binary) and one continuous, of the potential geographic distribution of the maned sloth. We defined the potential geographic distribution as the area where at least one model, after thresholding, predicted presence.

### Environmental variables

To investigate the importance of our environmental variables in determining species presence, we compared rescaled, standardized values of the environmental variables at locations where the species was predicted present or absent. To do this, we overlapped the layer of the single binary ensemble with the nine environmental variables used in the modeling. For each variable, we extracted the value for each pixel at which the species was predicted present or not present. To compare niche dimensions, we converted all variables to the same scale, such that the values from the whole study area ranged between 0 and 1. We assume that the most limiting variable for species occurrence is the one with the smallest standard deviation for predicted presence and with the greatest difference between the means of predicted present and not present.

To complement the previous analysis, we overlapped the maned sloth 42 occurrence points with the nine environmental variables used in the modeling, and extracted the value for each pixel. Then we performed a principal component analysis (PCA) to associate the maned sloth occurrence points records to the environmental variables, aiming to provide the means to explain the variance magnitudes related to environmental variables to reveal the internal structure of the data. This technique is used to represent, in the environmental space, the niche occupied by the species [56].

### Suitable forest fragments

To determine the total area of the most suitable forest fragments for the maned sloth we used the forest cover remnants of the Atlantic Forest (Figure 1) from Fundação SOS Mata Atlântica [32] and information about the Atlantic Forest vegetation types from IBGE (Instituto Brasileiro de Geografia e Estatística) [57], [58]. We calculated the area of each forest fragment within the potential geographic distribution area of the maned sloth, and considering that maned sloths have a home range varying from 0.008 to 0.16 km^2^ [22], [30], [38], and that some individuals have been sighted in forest fragments as small as 0.2 km^2^ [59], we selected forest fragments applying a buffer around those larger than 0.1 km^2^ within the continuous ensemble model of potential distribution. Areas were calculated in km^2^ using GIS and South America Albers Equal Area Conic projection.

The continuous ensemble model of potential geographic distribution in combination with the forest fragment layer represent the realized distribution more consistent with the current reality because of the maned sloth’s dependence on forest cover. Federal and state Brazilian strictly protected areas (information from CNUC/MMA http://mapas.mma.gov.br/i3geo/datadownload.htm), which is equivalent to the Strict Nature Reserves category of the International Union for Conservation of Nature, were overlapped with the thresholded forest fragments layer in order to produce a map indicating protected forests of a minimum size for maned sloths. We assumed that habitat with importance values higher than 67% have more probability to be strongholds areas, and habitat lower than 48% have less probability to be strongholds areas (natural breaks in ArcGIS was used to define the importance values). Between these two values, the probability is medium. To define a species stronghold we used several criteria: known presence of maned sloth within protected areas, forest fragments of minimum size, and high predicted suitability. Recommended survey areas were also suggested where there was high bioclimatic suitability but no known presence or absence data.

## Results

### Potential geographic distribution and habitat suitability

**Figure 2:**
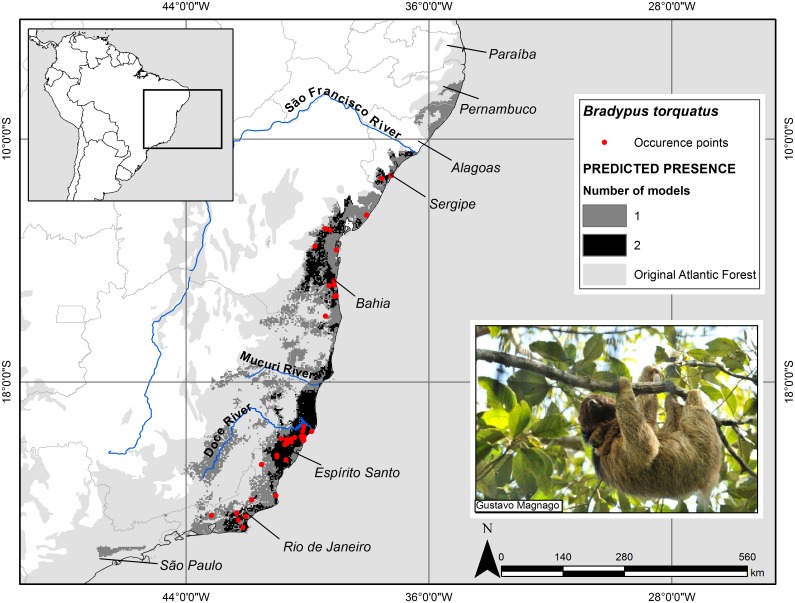
Potential geographic distribution of the maned sloth (*Bradypus torquatus*) in the Atlantic Forest. The potential geographic distribution of the maned sloth in the Atlantic Forest based on the binary ensemble model of Maxent and Mahalanobis Distance algorithm. The occurrence points used in the modeling are also shown in the map. Cartographic base: [79], Geographic Projection: Datum WGS 1984.

**Figure 3:**
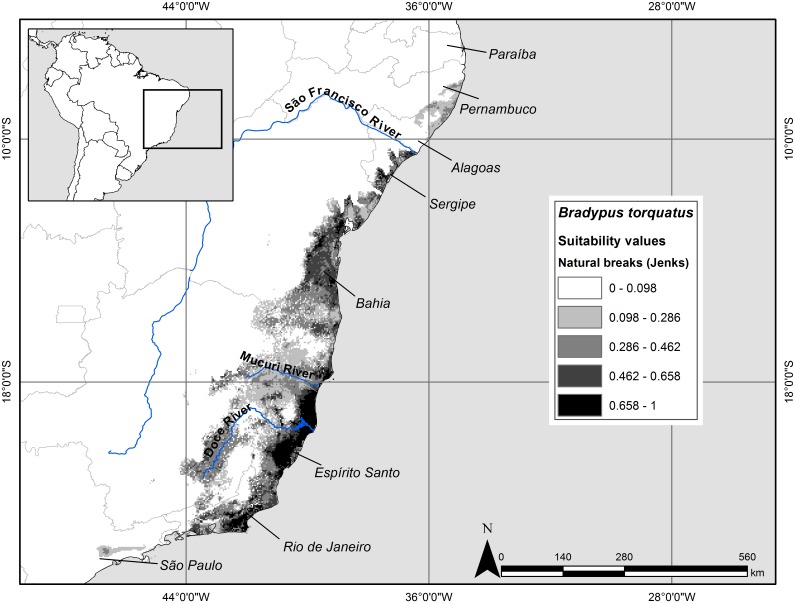
Potential geographic distribution with continuous values of the maned sloth in the Atlantic Forest. Potential geographic distribution of the maned sloth in the Atlantic Forest based on a continuous ensemble prediction. The occurrence points used in the modeling are also shown in the map. Cartographic base: [79]. Geographic Projection: Datum WGS 1984.

In general, the ensemble predictions showed that suitable habitat for maned sloth is restricted to the coastline of Brazil. The states of Sergipe, Bahia, Espírito Santo, and Rio de Janeiro were well-predicted by the intersection of the two models (Figure 2 and Figure 3). In Espírito Santo and southern Bahia the model reached more inland vegetation zones. The two areas considered as a distributional gap, between Mucuri and Doce Rivers, and in northern Rio de Janeiro, were predicted to be suitable by the model.

Both ensemble models predicted the southernmost suitable areas in the state of Rio de Janeiro and the northernmost areas surpassing São Francisco River, and reaching the state of Pernambuco. The continuous suitability map (Figure 3) showed high probabilities of suitable conditions (>0.658) in some parts of lowlands and highlands of Rio de Janeiro, south-center mountains and east-center coast of Espírito Santo. The lowest probabilities of suitable conditions (<0.462) occurred in the extreme northern coast of Atlantic Forest, interior of Bahia, state of Minas Gerais, and interior of Rio de Janeiro. In the state of the Espírito Santo, the area of suitability decreased mainly in the northwest of the north of Doce River, up to southern Bahia (Figure 3). In this state, the suitability values varied considerably in the south and it did not show a spatial pattern, although the values tended to increase toward the northeast. In Sergipe the probabilities of suitable conditions were predominantly medium (between 0.462 and 0.658).

### Model evaluation

Our predictions of the Maxent and Mahalanobis Distance models, separately, were highly significant (Figure S1 and S2). The average predictive ability of the partitioned models obtained by the ROC plot method was high, and very similar for both methods (Maxent AUC = 0.95 [SD = 0.02]; Mahalanobis Distance AUC = 0.95 [SD = 0.02]). In the same way, the models accuracy evaluated by the TSS were good for Mahalanobis Distance (0.724+/−SD 0.049), Maxent (0.796+/−SD 0.053), and for the ensemble (0.803).

### Environmental variables

**Figure 4:**
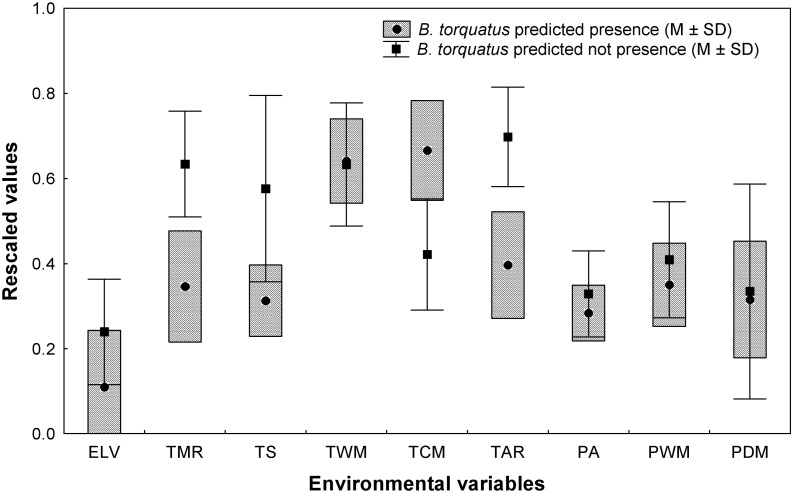
Rescaled environmental variables, representing predicted and not predicted values of presence of the maned sloth. Standardization of environmental variables to the same scale (0 to 1), based on the potential geographic distribution modeling for the Atlantic Forest. The whiskers represent the mean ± standard deviation of the pixels from areas that the model did not predict the presence of maned sloth. The boxes represent the mean ± standard deviation of the pixels from predicted suitable areas for the presence of maned sloth. Elevation (ELV), mean monthly temperature range (TMR), temperature seasonality (TS), maximum temperature of warmest month (TWM), minimum temperature of coldest month (TCM), temperature annual range (TAR), annual precipitation (PA), precipitation of wettest month (PWM) and precipitation of driest month (PDM).

We describe the habitat suitability of maned sloth as between 0 and 2,349.86 m of altitude, with an annual temperature between 8.5 and 19.4°C, and an annual precipitation between 753.01 and 2,592.0 mm. Table 1 shows the mean, maximum and minimum values of the environmental variables of suitable areas for *B. torquatus*, and table 2 shows the values for the most limiting environmental variables in areas of high probability of suitable conditions (Threshold>0.658). Comparison of environmental variables’ values among areas predicted as suitable and unsuitable reveals that temperature variation (such as monthly temperature range, seasonality, and annual range) presented quite-distinct values, showing that those variables are important to describe the climatic suitable areas for the maned sloth (Figure 4).

**Table 1:** Descriptive statistics of each environmental variable from suitable predicted pixels predicted by the binary ensemble model of the potential geographic distribution of the maned sloth (*Bradypus torquatus*).

Environmental variable	Mean	SD	Minimum	Maximum
Elevation (m)	337.1	211.3	0.0	2,108.8
Mean monthly temperature range (°C)	9.3	1.6	6.2	12.7
Temperature seasonality (K)	166.1	29.7	96.9	239.0
Maximum temperature of warmest month (°C)	29.7	1.7	20.4	33.4
Minimum temperature of coldest month (°C)	15.3	2.8	3.5	20.2
Temperature annual range (°C)	14.4	2.4	9.6	19.4
Annual precipitation (mm)	1,204.8	246.0	676.0	2,281.0
Precipitation of wettest month (mm)	181.8	44.2	76.0	321.0
Precipitation of driest month (mm)	43.6	22.0	9.0	136.0

**Table 2:** Descriptive statistics of the most limiting environmental variables for maned sloth (*Bradypus torquatus*) in areas of high probability of suitable conditions (Threshold>0.658), predicted by the continuous ensemble model of the potential geographic distribution.

Environmental variable	Mean	SD	Minimum	Maximum
Temperature seasonality (K)	170.4	33.6	94.3	280.7
Mean monthly temperature range (°C)	9.6	1.8	5.7	12.7
Minimum temperature of coldest month (°C)	15.0	3.0	0.7	21.3
Temperature annual range (°C)	14.8	2.8	8.5	20.3

**Figure 5:**
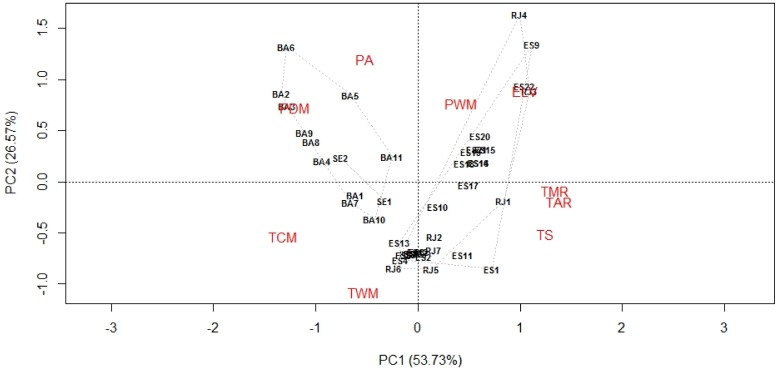
Projection of the nine environmental variables and the maned sloth points records on the first and second factor planes. Principal component analysis (PCA) of the association of the maned sloth points records and the nine environmental variables used in the distribution modelling procedure. Sergipe (SE), Bahia (BA), Espírito Santo (ES), Rio de Janeiro (RJ). Elevation (ELV), mean monthly temperature range (TMR), temperature seasonality (TS), maximum temperature of warmest month (TWM), minimum temperature of coldest month (TCM), temperature annual range (TAR), annual precipitation (PA), precipitation of wettest month (PWM) and precipitation of driest month (PDM). Points records localities are available (Table S1).

To identify the spatial correlation of maned sloth occurrence points and the environmental variables through PCA, we observed that the two first axes explain more than 80% of the environmental data variation (Figure 5). The first axis (PC1) shows a latitudinal gradient, splitting two environments groups. The first one shows a population group located in the states of Rio de Janeiro and Espírito Santo, and the more important environment variables that explains the distribution are mean monthly temperature range, temperature annual range, temperature seasonality, precipitation of wettest month and elevation. The second group is formed by populations located in the states of Bahia and Sergipe, and the most important environmental variables associated are annual precipitation, precipitation of driest month, minimum temperature of coldest month, and maximum temperature of warmest month.

The second axis (PC2) shows an apparent altitudinal gradient, especially for the records located in Rio de Janeiro and Espírito Santo. The environmental variables associated with the high altitudes are precipitation of driest month, annual precipitation, precipitation of wettest month, and elevation. The lower altitudes are associated with minimum temperature of coldest month, maximum temperature of warmest month, mean monthly temperature range, temperature annual range, and temperature seasonality.

### Suitable forest fragments

We found 3,693 forest fragments with an area of 16,000.67 km^2^ within the potential geographic distribution (Table 3). The primary land cover within suitable habitat was Ombrophilous Dense Forest, which covered 9,795.82 km^2^, followed by Semi-deciduous Seasonal Forest, with 3,358.01. Most forest fragments were small and less than 5 km^2^ (85%). However, they also represent the class with the greatest total area (39%), followed by the class between 10 and 100 km^2^ (31% of the total area).

**Table 3:** Number of forest fragments (NF) and summed areas (SA) in km^2^ predicted by the potential geographic distribution models of maned sloth (*Bradypus torquatus*), according to vegetation type and area.

	1−5 km2	5−10 km2	10−100 km2	≥100 km2	Total
**Vegetation type**	NF (SA)	NF (SA)	NF (SA)	NF (SA)	NF (SA)
**Deciduous Seasonal Forest**	129 (266.82)	16 (113.81)	7 (155.42)	-	152 (536.05)
**Semi-deciduous Seasonal Forest**	943 (1,811.41)	69 (448.27)	31 (570.95)	2 (527.38)	1045 (3,358.01)
**Ombrophilous Open Forest**	187 (348.75)	14 (91.85)	7 (131.43)	-	208 (572.03)
**Ombrophilous Dense Forest**	1,547 (3,082.60)	199 (1,321.26)	132 (3,258.61)	12 (2,133.35)	1,890 (9,795.82)
**Others type of forest**	333 (677.93)	31 (207.93)	34 (852.90)	-	398 (1,738.76)
**Total**	3,139 (6,187.51)	329 (2,183.12)	211 (4,969.31)	14 (2,660.73)	3693 (16000.67)

**Figure 6:**
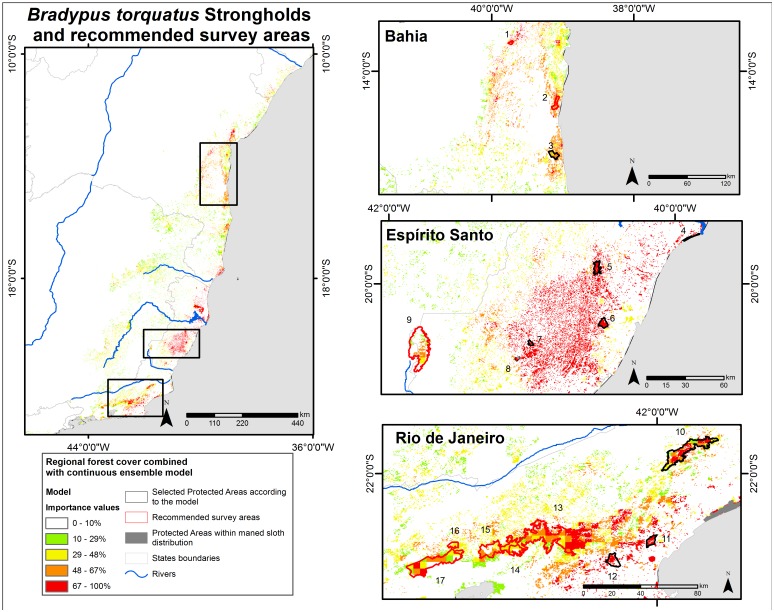
Maned sloth’s (*Bradypus torquatus*) strongholds and recommended survey areas. Current fragments of the Atlantic Forest, combined with the potential geographic distribution modeling and the federal and state strictly protected areas. We used each fragment larger than 0.1 km^2^ to build a buffer. Protected areas: 1. Estação Ecológica de Wenceslau Guimarães, 2. Parque Estadual Serra do Conduru, 3. Reserva Biológica de Una, 4. Reserva Biológica de Comboios, 5. Reserva Biológica Augusto Ruschi, 6. Reserva Biológica de Duas Bocas, 7. Parque Estadual de Pedra Azul, 8. Parque Estadual do Forno Grande, 9. Parque Nacional do Caparaó, 10. Parque Estadual do Desengano, 11. Reserva Biológica União, 12. Reserva Biológica Poço das Antas, 13. Parque Estadual dos Três Picos, 14. Estação Ecológica Estadual do Paraíso, 15. Parque Nacional Serra dos Órgãos, 16. Reserva Biológica de Araras, 17. Reserva Biológica do Tinguá. Cartographic bases: [32], [79], CNUC/MMA. Geographic Projection; South America Albers Equal Area Conic.

The suitable forest fragment model, indicated three regions with elevated forest remnants and high suitability: the central region of Rio de Janeiro, central Espírito Santo, and the central-coast of Bahia (Figure 6). With regard to the protected areas, 58 reserves overlap to the binary ensemble model of the maned sloth; however only 17 covering 2,122 km^2^ are located in areas with forest remnants and high suitability (importance value higher than 48%; Figure 6).

## Discussion

### Potential geographic distribution and habitat suitability

Our predicted potential geographic distribution of the maned sloth is similar to its documented range [27], [60], [61], but when compared with Fonseca & Aguiar [62], and more recently, with Hirsch and Chiarello [21], there are some important differences. First, the models do not indicate a climatic relation with the absence of the species in between Rio de Janeiro and Espírito Santo, or between Espírito Santo and Bahia. Second our ensemble model also indicated suitable climatic conditions in the southern Atlantic Forest region (in São Paulo), and northern Atlantic forest region (in Alagoas, Pernambuco and Paraíba), areas where the species is supposedly absent (see [20], [21], [27], [62], [63]). Scientists have long discussed the historic presence of maned sloths in the northern Atlantic [21], [64] however, regardless of if it was previously present, it is clear now that the maned sloth no longer exist in those areas.

Currently, confirmed records of remaining populations of maned sloth come from Bahia, Espírito Santo, and Rio de Janeiro [28]. Until recently, it was thought that the species was no longer in Sergipe because of deforestation [63], but recent evidence confirmed the presence of *B*. *torquatus* in this state [65], drawing new attention to this region.

Our binary ensemble of potential geographic distribution model, based mainly on climatic conditions, did not reconstruct the apparent gap in the distribution of the maned sloth between Doce River, in northern Espírito Santo, and Mucuri River, in southern Bahia, and also between southern Espírito Santo and northern Rio de Janeiro. Rather, the binary model indicates these areas are climatically suitable (Figure 2 and Figure 3). If the model is correct, then factors other than current climate may be responsible for the absence of species in that area, such as historical process of isolation associated with climatic and consequent vegetation changes and retractions that occurred along the Quaternary. The reduction and expansion of forested areas could be closely related to sloth dispersal, since sloths have a strictly arboreal habit [60], [66].

On the other hand, the continuous ensemble potential model (Figure 3) suggests that suitability is lower between southern Espírito Santo and northern Rio de Janeiro than other areas, despite it still indicate predicted presence. But within the gap between Espírito Santo and Bahia the probability of suitable conditions continued higher on the region of Doce River mouth and it decreased to the interior. These lowest values of suitability can mean that the level of environmental conditions required for the maned sloth to survive is higher in areas with lower values of environmental suitability.

The gaps in those regions may be related to the prevalence of a dryer forest [21], [63]. In both gap regions, the vegetation type is called lowland forest on tertiary “tabuleiros” [67]–[70]. The topography of those regions is flat over large areas, not exceeding 200 m altitude [68], [71]. The lowland forests are distinguished from other formations of the Atlantic Forest because they occupy a large area of coastal plain, from tertiary origin, with its flora species distributed along a climate gradient (direction coastal-interior) [69]. Additionally, the interior forest presents relatively little understory vegetation and more epiphytic species [67]. Both the northern Espírito Santo and southern Bahia regions have the same vegetation cover class (Ombrophilous Dense Forest) as the other areas known for maned sloth [72], [73]. But some authors consider it as a Lowland Semi-deciduous Seasonal Forest [69], [70], as well as the gap between southern Espírito Santo and northern Rio de Janeiro [72]. Other endemic mammals, such as the Thin-spined Porcupine, *Chaetomys subspinosus* (Olfers, 1818), and the northern muriqui, *Brachyteles hypoxanthus* (Kuhl, 1820), have the same interesting gap in their distributions between the northern Espírito Santo and Southeastern Bahia [20].

### Environmental variables

Our binary ensemble model yielded a distribution of *B. torquatus* close to that proposed by Oliver and Santos [20], supporting the idea that the range of maned sloth distribution is strongly influenced by climatic factors, mainly temperature, and, indirectly elevation. Surface temperature is suspected as the major factor behind the apparent change in the activity period of maned sloths [30], [38], particularly extreme low temperatures [74].

Our comparisons of standardized variables supports these ideas with the result that environmental variables related to the variability of temperature, annually or monthly, are the most limiting factors for the occurrence of the maned sloth. In other words, suitable regions are those that present a lower variation in temperature during the year or month (Figure 4). This is reasonable for an animal that has adapted to the environment by maximizing their activity in periods with more favorable temperatures, increasing their diurnal activity in colder regions or seasons or their nocturnal activities in warmer regions or seasons [38]. This means that they behaviorally thermoregulate their body temperature by moving in the canopy to places with more or less sunlight [30]. The variable ‘minimum temperature of coldest month’ was also a limiting factor for the species, restricting its modeled distribution to regions that did not present extreme low temperatures in the winter.

When only the records points are taken into account, environmental conditions may vary spatially and with the altitude (Table S2). According to our analysis, two climatically different populations are present in the Atlantic Forest: a southern maned sloth population (represented by records from Rio de Janeiro and Espírito Santo), and a northern one (represented by records from Bahia and Sergipe). Previous works [66], [75] has recognized at least two major phylogeographic groups of *B. torquatus*, representing a north and south divergence, separated by the gap between Mucuri River and Doce River (Figure 1). Because terrain configuration varies more in the south, temperature range, elevation and precipitation from the wettest month are the main environmental variables associated with the maned sloth points records. In the north, because of high temperatures and humidity, precipitation and extreme temperatures are the most important variables associated with the maned sloth points records.

Maned sloth presence can also be related to altitude, although this association is not so evident for the northern populations. Again, probably because of the terrain configuration, elevation and precipitation are the most important environmental variables in high altitudes associated with maned sloth records. In low altitudes, temperature is the main variable related with maned sloth’s presence.

### Suitable forest fragments and conservation areas for *Bradypys torquatus*

Records of maned sloth from museums and literature are almost exclusively from Ombrophilous Dense Forest. This forest predominates, both in number and area of patches (Table 3). Hirsch & Chiarello also supported that the majority of confirmed records of maned sloth are within the zone of Ombrophilous Dense Forest. Within distributional areas of maned sloth, individuals of this species use both primary and secondary forests, but it is difficult for them to exist in more open vegetation [22]. However, because the Atlantic forest has undergone severe deforestation, remaining Ombrophilous Dense Forest is strongly fragmented. And, despite numerous suitable fragments in the potential geographic distribution, (Table 3) maned sloth occurs only in a small fraction of these areas [63].

The stronghold areas for maned sloths are in mountainous region of Rio de Janeiro, central Espírito Santo and coastal regions of southern Bahia (Figure 6), because of climatic suitability (considering the environmental variables of Table 1 and Figure 3), high forest patch density and size, presence of the largest known populations, and also the presence of federal and state protected areas. The region of Sergipe was not indicated as a stronghold, mostly because there are so few forest fragments and protected areas. However, it is the northernmost presence of maned sloth, and because the recently discovered population is threatened to vanish with the forests, more attention is needed in the region. The discovery of an unknown population in Sergipe increases the expectation of finding more populations in the Atlantic Forest, and should elicit increased research and conservation planning for this species. We acknowledge that the term “stronghold” here merely represents our definition, which are the most important areas for conservation purposes.

Confirmed records of maned sloths (Table S1) are present in six protected areas that, together, have an area of 1,200 km^2^: Reserva Biológica de Una (Bahia), Reserva Biológica Augusto Ruschi (Espírito Santo), Parque Estadual de Pedra Azul (Espírito Santo), Parque Estadual do Desengano (Rio de Janeiro) Reserva Biológica União (Rio de Janeiro), Reserva Biológica Poço das Antas (Rio de Janeiro). Eleven other protected areas have high suitability values and could be important reserves for the maned sloth (see Figure 6). Those protected areas are: Estação Ecológica de Wenceslau Guimarães (Bahia) Parque Estadual Serra do Conduru (Bahia), Reserva Biológica de Comboios (Espírito Santo), Reserva Biológica de Duas Bocas (Espírito Santo), Parque Estadual do Forno Grande (Espírito Santo), Parque Nacional do Caparaó (Espírito Santo/Minas Gerais), Parque Estadual dos Três Picos (Rio de Janeiro), Estação Ecológica Estadual do Paraíso (Rio de Janeiro), Parque Nacional Serra dos Órgãos (Rio de Janeiro), Reserva Biológica de Araras (Rio de Janeiro), Reserva Biológica do Tinguá (Rio de Janeiro).

The conservation outlook of the maned sloth is not altogether bleak. While present in most of the protected areas in the region, the maned sloth is still susceptible to its primary threats and deforestation. Taking this into consideration, the 6 known protected areas and 11 suspected protected areas (in sum 2,122.79 km^2^) have great potential to be considered management areas for maned sloths, and they represent a total of 29% of all reserves located within the maned sloth’s potential geographic distribution areas. But it has also to be considered that only 21% of the Ombrophilous Dense Forest in the potential geographic distribution is protected by the federal and state Brazilian strictly protected areas network, which limits the conservation actions for maned sloth. Yet worryingly, over 1/3^rd^ the total area of forest fragments of suitable habitat and the greatest number of forest fragments of suitable habitat is from the category of smallest fragment size (1–5 km). This suggests that lots more habitats may be easily lost in the next years. Therefore, not only conservation actions should be taken in the protected areas, but also they are dearly needed to restore and reconnect the surrounding habitats and other important ones where maned sloth is known.

Distribution models can be used to identify areas that are important for species of conservation concern [7]. Here we indicate several species stronghold areas and recommended survey areas. In addition, we identified the network of protected areas to be considered in the maned sloth management. This is an efficient approach with good results to be used in endangered species management and conservation. Our results can be applied for novel surveys and discovery of unknown populations, and help the selection of priority areas for management and conservation planning [76]–[78], especially of rare and relatively cryptic species directed associated with forested habitats.

## Supporting Information

Figure S1 **Binary models of potential geographic distribution for the maned sloth (** ***Bradypus torquatus*** **).** The models represents the results from Maxent (on the left) and Mahalanobis Distance (on the right) algorithms. The areas with low and high suitability values are indicated as 0 and 1 for each algorithm model. Cartographic bases: [79]. Geographic Projection; Datum WGS 1984.
(TIF)

Figure S2 **Continuous models of potential geographic distribution for the maned sloth (** ***Bradypus torquatus*** **).** The models represents the results from Maxent (on the left) and Mahalanobis Distance (on the right) algorithms. The areas with low and high suitability values are scaled from 0 to 1 for each algorithm model. Cartographic bases: [79]. Geographic Projection; Datum WGS 1984.
(TIF)

Table S1 **Maned sloth (** ***Bradypus torquatus*** **) presence points used in modeling.** This table gives the GPS coordinates, localities, and sources for the Maned sloth presence data used in the analysis. Sergipe (SE), Bahia (BA), Espírito Santo (ES), Rio de Janeiro (RJ).
(DOC)

Table S2 **Environmental variables values for the 42 presence points of maned sloth.** The values are from each environmental variable pixel coincident with the records points of the maned sloth (*Bradypus torquatus*). Sergipe (SE), Bahia (BA), Espírito Santo (ES), Rio de Janeiro (RJ). Elevation (ELV), mean monthly temperature range (TMR), temperature seasonality (TS), maximum temperature of warmest month (TWM), minimum temperature of coldest month (TCM), temperature annual range (TAR), annual precipitation (PA), precipitation of wettest month (PWM) and precipitation of driest month (PDM). Points records localities are available (Table S1).
(DOC)
