# Supplementary figures and images for: The Distributional Ecology of the Maned Sloth: Environmental Influences on Its Distribution and Gaps in Knowledge
Source: PLoS One. 2014 Oct 22;9(10):e110929. doi: 10.1371/journal.pone.0110929 (PMC4206454; doi:10.1371/journal.pone.0110929)

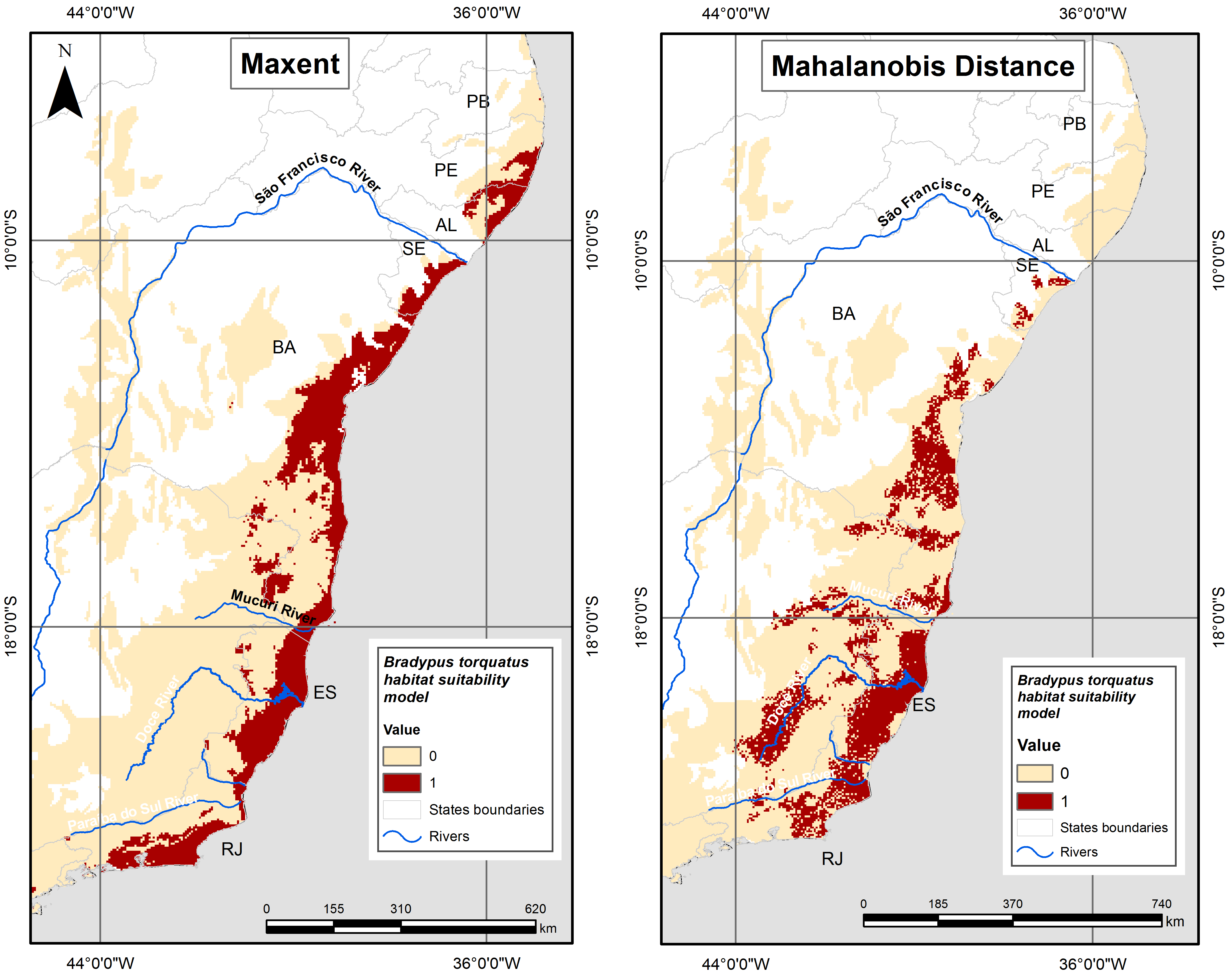

Supplement: Figure S1 — Binary models of potential geographic distribution for the maned sloth ( Bradypus torquatus ). The models represents the results from Maxent (on the left) and Mahalanobis Distance (on the right) algorithms. The areas with low and high suitability values are indicated as 0 and 1 for each algorithm model. Cartographic bases: [79]. Geographic Projection; Datum WGS 1984. (TIF) [file pone.0110929.s001.tif]

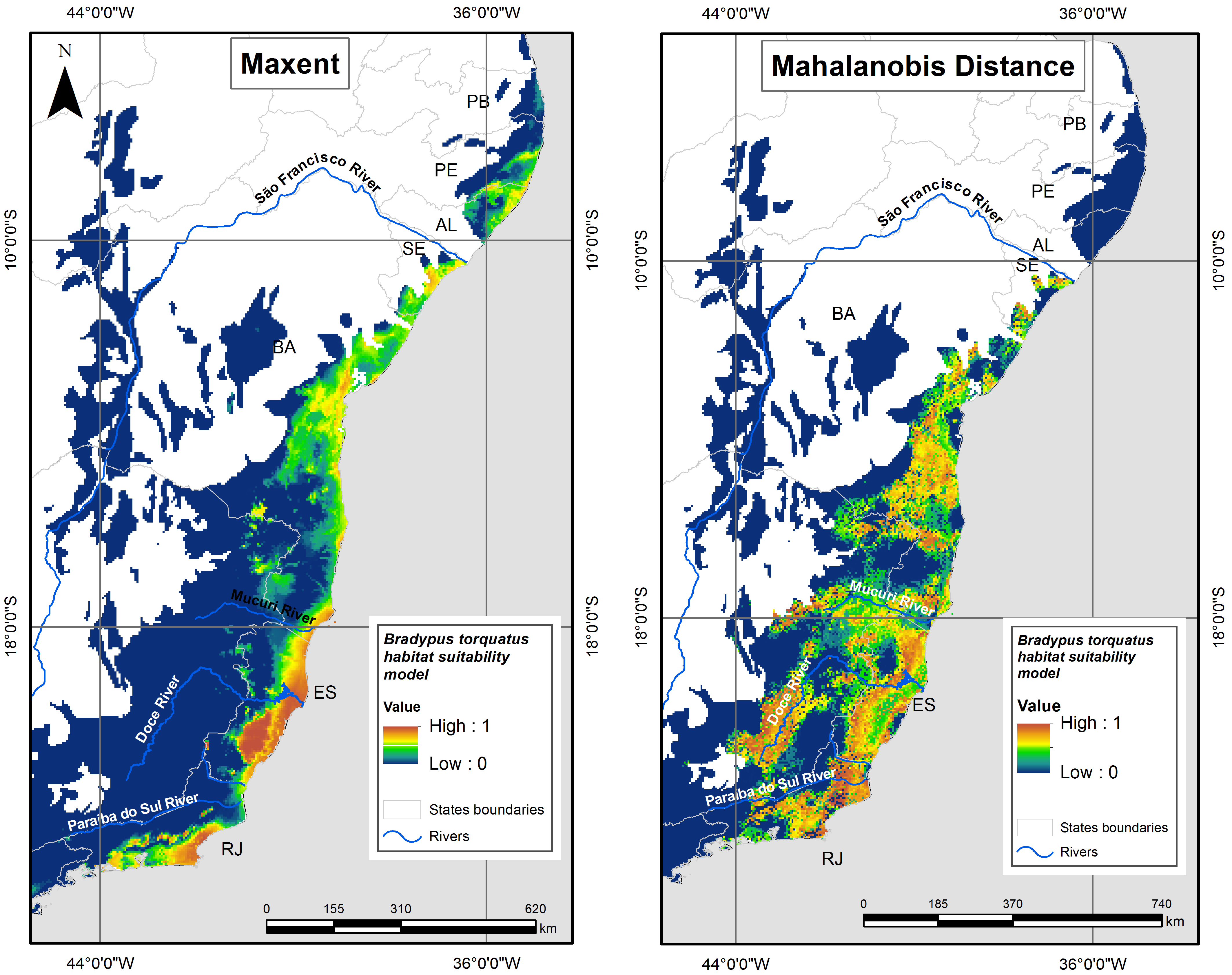

Supplement: Figure S2 — Continuous models of potential geographic distribution for the maned sloth ( Bradypus torquatus ). The models represents the results from Maxent (on the left) and Mahalanobis Distance (on the right) algorithms. The areas with low and high suitability values are scaled from 0 to 1 for each algorithm model. Cartographic bases: [79]. Geographic Projection; Datum WGS 1984. (TIF) [file pone.0110929.s002.tif]
