# Supplementary material for: The Distributional Ecology of the Maned Sloth: Environmental Influences on Its Distribution and Gaps in Knowledge
Source: PLoS One. 2014 Oct 22;9(10):e110929. doi: 10.1371/journal.pone.0110929 (PMC4206454; doi:10.1371/journal.pone.0110929)
Supplement: Table S1 — Maned sloth ( Bradypus torquatus ) presence points used in modeling. This table gives the GPS coordinates, localities, and sources for the Maned sloth presence data used in the analysis. Sergipe (SE), Bahia (BA), Espírito Santo (ES), Rio de Janeiro (RJ). (DOC) [file pone.0110929.s003.doc]

**Table S1. Maned sloth (*Bradypus torquatus*)** **presence points used in modeling.** This table gives the GPS coordinates, localities, and sources for the Maned sloth presence data used in the analysis. Sergipe (SE), Bahia (BA), Espírito Santo (ES), Rio de Janeiro (RJ).

| **Record** | **Code** | **Locality** | **Municipality** | **State** | **Latitude** | **Longitude** | **Reference** |
| --- | --- | --- | --- | --- | --- | --- | --- |
| 1 | BA1 | Vicinity of Santa Maria Eterna | Belmonte | BA | -15.83300 | -39.40000 |  |
| 2 | BA2 | Fazenda Pirataquisse, Banco da Vitória | Ilhéus | BA | -14.80000 | -39.11660 | MNRJ (11206); |
| 3 | BA3 | Urucutuca, Aritaguá | Ilhéus | BA | -14.65778 | -39.12528 | MNRJ (11395, 23896); |
| 4 | BA4 | Vicinity of Itabuna | Itabuna | BA | -14.83333 | -39.28333 |  |
| 5 | BA5 | Reserva Camurujipe/Passagem Grande | Mata de São João | BA | -12.50000 | -38.05000 |  |
| 6 | BA6 | Restinga de Pratigi | Nilo Peçanha | BA | -13.65000 | -39.04000 |  |
| 7 | BA7 | Vicinity of Santo Antônio de Jesus | Santo Antônio de Jesus | BA | -13.00000 | -39.25000 |  |
| 8 | BA8 | Reserva Biológica de Una | Una | BA | -15.17714 | -39.10497 |  |
| 9 | BA9 | Ecoparque de Uma | Una | BA | -15.16311 | -39.05180 |  |
| 10 | BA10 | Vicinity of São Miguel das Matas | Varzedo | BA | -12.95000 | -39.41667 |  |
| 11 | BA11 | Fazenda Piabanha, Três Braços | Wenceslau Guimarães | BA | -13.53333 | -39.75000 | USNM (uncatalogued); |
| 12 | ES1 | Engenheiro Reeve | Alegre | ES | -20.71916 | -41.51221 | ZSM (1281) |
| 13 | ES2 | Vicinity of Sagrada Família | Alfredo Chaves | ES | -20.56667 | -40.70000 |  |
| 14 | ES3 | Private forest fragment | Aracruz | ES | -19.79000 | -40.20000 |  |
| 15 | ES4 | Private forest fragment 1 - Aracruz Cellulose | Aracruz | ES | -19.93333 | -40.13333 |  |
| 16 | ES5 | Private forest fragment 2 - Aracruz Cellulose | Aracruz | ES | -19.91667 | -40.11667 |  |
| 17 | ES6 | Private forest fragment 3 - Aracruz Cellulose (M7/317) | Aracruz | ES | -19.80000 | -40.11667 |  |
| 18 | ES7 | Private forest fragment 4 and 5 - Aracruz Cellulose | Aracruz | ES | -19.61667 | -40.15000 |  |
| 19 | ES8 | Putiri | Aracruz | ES | -19.87743 | -40.16108 |  |
| 20 | ES9 | Parque Estadual da Pedra Azul | Domingos Martins | ES | -20.41615 | -41.00974 |  |
| 21 | ES10 | Pau Gigante | Ibiraçu | ES | -19.83473 | -40.41806 | MNRJ (23921); |
| 22 | ES11 | Private forest fragment | Itarana | ES | -19.87000 | -40.87000 |  |
| 23 | ES12 | Bebedouro | Linhares | ES | -19.46809 | -40.11878 | MEL (M007); |
| 24 | ES13 | Regência | Linhares | ES | -19.62792 | -39.85978 | MBML (379) |
| 25 | ES14 | Private forest fragment | Santa Maria de Jetibá | ES | -20.03333 | -40.68333 |  |
| 26 | ES15 | Rio das Pedras | Santa Maria de Jetibá | ES | -20.06209 | -40.74414 |  |
| 27 | ES16 | Sítio de Paulo Seick | Santa Maria de Jetibá | ES | -20.04194 | -40.70224 |  |
| 28 | ES17 | Alto das Tabocas | Santa Teresa | ES | -19.90531 | -40.69428 | MNRJ (5632); |
| 29 | ES18 | Estação Biológica de Santa Lúcia | Santa Teresa | ES | -19.97323 | -40.53047 | MBML (281); |
| 30 | ES19 | Reserva Biológica Augusto Ruschi/Nova Lombardia | Santa Teresa | ES | -19.88866 | -40.54556 | MBML (2798); |
| 31 | ES20 | Parque Natural Municipal de São Lourenço | Santa Teresa | ES | -19.92452 | -40.62348 |  |
| 32 | ES21 | Urban zone | Santa Teresa | ES | -19.93773 | -40.59414 |  |
| 33 | ES22 | Hotel Fazenda Monte Verde | Vargem Alta | ES | -20.46464 | -41.00227 | UFPB (409) |
| 34 | RJ1 | Parque Estadual do Desengano | Campos dos Goytacazes | RJ | -21.88220 | -41.83154 |  |
| 35 | RJ2 | Reserva Biológica União | Casimiro de Abreu | RJ | -22.42465 | -42.03805 |  |
| 36 | RJ3 | Serra de Macaé | Nova Friburgo | RJ | -22.31667 | -42.33333 | MZUSP (2806); |
| 37 | RJ4 | - | Petrópolis | RJ | -22.39786 | -43.15712 | NMW (2016/ST 284A) |
| 38 | RJ5 | Ilha Brussaí [Gruçaí] | São João da Barra | RJ | -21.73639 | -41.03558 | MZUSP (10074); |
| 39 | RJ6 | - | São Pedro da Aldeia | RJ | -22.78576 | -42.12375 |  |
| 40 | RJ7 | Reserva Biológica de Poço das Antas | Silva Jardim | RJ | -22.54937 | -42.27660 |  |
| 41 | SE1 | Fazenda Riacho Seco | Arauá | SE | -11.30000 | -37.55000 |  |
| 42 | SE2 | Fazenda Trapsa | Itaporanga d'Ajuda | SE | -11.20000 | -37.23333 |  |

BA, Bahia; ES, Espírito Santo; RJ, Rio de Janeiro; SE, Sergipe; MNRJ, Museu Nacional do Rio de Janeiro; USNM, United States Natural Museum; ZSM, Zoologische Staatssammlung München; MEL, Museu Elias Lorenzute; MBML, Museu de Biologia Mello Leitão; UFPB, Universidade Federal da Paraíba; MZUSP, Museu de Zoologia da Universidade de São Paulo; NMW, Naturhistorisches Museum Wien

**References**

Oliver WLR and Santos FB (1991) Threatened endemic mammals of the Atlantic forest region of south-east Brazil. Jersey: Jersey Wildlife Preservation Trust. 126 p.

Vaz SM (2003) A localidade tipo da preguiça-de-coleira, *Bradypus torquatus* Illiger, 1811 (Xenarthra, Bradypodidae). Edentata 5: 1–4.

Vaz SM (2003) Lista de localidades de capturas de Xenartros sob ameaça de extinção no Brasil. Edentata 5: 4–5.

Vaz SM (2005) Mamíferos colecionados pelo serviço de estudos e pesquisas sobre a febre amarela nos municípios de Ilhéus e Buerarema, estado da Bahia, Brasil. Arq Museu Nac 63: 21–28.

Anderson RP and Handley CO (2001) A new species of three-toed sloth (Mammalia: Xenarthra) from Panama, with a review of the genus *Bradypus*. P Biol Soc Wash 114: 1–33.

Lara-Ruiz P, Chiarello AG and Santos FR (2008) Extreme population divergence and conservation implications for the rare endangered Atlantic Forest sloth, *Bradypus torquatus* (Pilosa: Bradypodidae). Biol Conserv 141: 1332–1342.

Lara-Ruiz P and Chiarello AG (2005) Life-history traits and sexual dimorphism of the Atlantic forest maned sloth *Bradypus torquatus* (Xenarthra: Bradypodidae). J Zool, Lond 267: 63–73.

Chiarello AG (1999) Effects of fragmentation of the Atlantic forest on mammal communities in south-eastern Brazil. Biol Conserv 89: 71–82.

Pinder L (1986) Conservation of the maned sloth (*Bradypus torquatus*). Washington: Report to World Wildlife Fund. 68 p.

Lorenzutti R and Almeida AP (2006) A coleção de mamíferos do Museu Elias Lorenzutti em Linhares, Estado do Espírito Santo, Brasil. Bol Mus Biol Mello Leitão 19: 59–74.

Nunes SF (2004) Riqueza e abundância de mamíferos de médio e grande porte em uma paisagem fragmentada na região serrana do Espírito Santo, Brasil. Departamento de Biologia. Vitória: Universidade Federal do Espírito Santo. pp. 65.

Dias BB, Santos LAD, Lara-Ruiz P, Cassano CR, Pinder L, et al. (2008) First observation on mating and reproductive seasonality in maned sloths *Bradypus torquatus* (Pilosa: Bradypodidae). J Ethol 26.

Passamani M, Mendes SL and Chiarello AG (2000) Non-volant mammals of the Estação Biológica de Santa Lúcia and adjacent areas of Santa Teresa, Espírito Santo, Brazil. Bol Mus Biol Mello Leitão 11: 201–214.

Chiarello AG (1998) Diet of the Atlantic forest maned sloth *Bradypus torquatus* (Xenarthra: Bradypodidae). J Zool 246: 11–19.

Chiarello AG (1998) Activity budgets and ranging patterns of the Atlantic forest maned sloth *Bradypus torquatus* (Xenarthra: Bradypodidae). J Zool 246: 1–10.

Chiarello AG, Chivers DJ, Bassi C, Maciel MAF, Moreira LS, et al. (2004) A translocation experiment for the conservation of maned sloths, *Bradypus torquatus* (Xenarthra, Bradypodidae). Biol Conserv 118: 421–430.

Srbek-Araujo AC and Chiarello AG (2005) Is camera-trapping an efficient method for surveying mammals in Neotropical forests? A case study in south-eastern Brazil. J Trop Ecol 21: 121–125.

Chiarello AG (2001) A translocation experiment for the conservation of maned sloths (*Bradypus torquatus*), a species threatened with extinction in the Brazilian Atlantic forest. Edentata 4: 23-25.

Wetzel RM and Ávila-Pires FD (1980) Identification and distribution of the recent sloths of Brazil (Edentata). Rev Bras Biol 40: 831-836.

Wied-Neuwied M (1989) Viagem ao Brasil. São Paulo: EDUSP.

Lisboa CV, Mangia RH, Lima RH, Martins A, Dietz J, et al. (2004) Distinct patterns of *Trypanosoma cruzi* infection in *Leontopithecus rosalia* in distinct Atlantic Coastal Rainforest fragments in Rio de Janeiro – Brazil. Parasitology 129: 703–711.

Chagas RRD, Souza-Alves JP, Jerusalinsky L and Ferrari S (2009) New records of *Bradypus torquatus* (Pilosa: Bradypodidae) from Southern Sergipe, Brazil. Edentata 8–10: 21–24.
