# Supplementary material for: The Distributional Ecology of the Maned Sloth: Environmental Influences on Its Distribution and Gaps in Knowledge
Source: PLoS One. 2014 Oct 22;9(10):e110929. doi: 10.1371/journal.pone.0110929 (PMC4206454; doi:10.1371/journal.pone.0110929)
Supplement: Table S2 — Environmental variables values for the 42 presence points of maned sloth. The values are from each environmental variable pixel coincident with the records points of the maned sloth (Bradypus torquatus). Sergipe (SE), Bahia (BA), Espírito Santo (ES), Rio de Janeiro (RJ). Elevation (ELV), mean monthly temperature range (TMR), temperature seasonality (TS), maximum temperature of warmest month (TWM), minimum temperature of coldest month (TCM), temperature annual range (TAR), annual precipitation (PA), precipitation of wettest month (PWM) and precipitation of driest month (PDM). Points records localities are available (Table S1). (DOC) [file pone.0110929.s004.doc]

**Table S2. Environmental variables values for the 42 presence points of maned sloth.** The values are from each environmental variable pixel coincident with the records points of the maned sloth (*Bradypus torquatus*). Sergipe (SE), Bahia (BA), Espírito Santo (ES), Rio de Janeiro (RJ). Elevation (ELV), mean monthly temperature range (TMR), temperature seasonality (TS), maximum temperature of warmest month (TWM), minimum temperature of coldest month (TCM), temperature annual range (TAR), annual precipitation (PA), precipitation of wettest month (PWM) and precipitation of driest month (PDM). Points records localities are available (Table S1).

| **Record** | **Code** | **Latitude** | **Longitude** | **ELV** | **PA** | **PWM** | **PDM** | **TMR** | **TS** | **TWM** | **TCM** | **TAR** |
| --- | --- | --- | --- | --- | --- | --- | --- | --- | --- | --- | --- | --- |
| 1 | BA1 | -15.833 | -39.400 | 160.980 | 1196.02 | 150.000 | 72.001 | 7.742 | 131.450 | 29.000 | 17.500 | 11.500 |
| 2 | BA2 | -14.800 | -39.117 | 58.998 | 1815.04 | 197.001 | 117.004 | 6.308 | 98.037 | 29.400 | 19.100 | 10.300 |
| 3 | BA3 | -14.658 | -39.125 | 13.009 | 1779.03 | 194.003 | 107.003 | 6.367 | 99.496 | 29.600 | 19.600 | 10.000 |
| 4 | BA4 | -14.833 | -39.283 | 81.991 | 1409.04 | 172.002 | 73.003 | 6.617 | 103.668 | 29.400 | 18.800 | 10.600 |
| 5 | BA5 | -12.500 | -38.050 | 60.007 | 1775.01 | 317.999 | 73.000 | 7.533 | 137.443 | 30.200 | 18.800 | 11.400 |
| 6 | BA6 | -13.650 | -39.040 | 58.983 | 2151.00 | 237.005 | 121.000 | 6.383 | 118.779 | 29.400 | 19.400 | 10.000 |
| 7 | BA7 | -13.000 | -39.250 | 202.988 | 1171.99 | 129.000 | 58.002 | 6.542 | 142.979 | 28.500 | 18.000 | 10.500 |
| 8 | BA8 | -15.177 | -39.105 | 73.997 | 1560.03 | 151.001 | 99.002 | 6.817 | 110.109 | 29.300 | 18.500 | 10.800 |
| 9 | BA9 | -15.163 | -39.052 | 63.997 | 1627.02 | 153.002 | 104.002 | 6.775 | 108.344 | 29.300 | 18.600 | 10.700 |
| 10 | BA10 | -12.950 | -39.417 | 382.934 | 954.97 | 113.000 | 40.996 | 6.583 | 145.217 | 27.400 | 16.900 | 10.500 |
| 11 | BA11 | -13.533 | -39.750 | 635.967 | 1006.01 | 109.003 | 53.000 | 7.567 | 134.156 | 26.500 | 15.100 | 11.400 |
| 12 | ES1 | -20.719 | -41.512 | 290.060 | 1147.02 | 205.001 | 22.000 | 11.792 | 202.410 | 31.600 | 13.300 | 18.300 |
| 13 | ES2 | -20.567 | -40.700 | 526.954 | 1216.99 | 190.999 | 42.000 | 9.458 | 178.494 | 29.200 | 14.000 | 15.200 |
| 14 | ES3 | -19.790 | -40.200 | 42.994 | 1169.00 | 189.000 | 37.000 | 8.925 | 179.830 | 31.700 | 17.100 | 14.600 |
| 15 | ES4 | -19.933 | -40.133 | 33.995 | 1149.00 | 186.000 | 41.000 | 8.475 | 179.551 | 31.500 | 17.500 | 14.000 |
| 16 | ES5 | -19.917 | -40.117 | 19.000 | 1148.00 | 184.000 | 42.000 | 8.383 | 180.391 | 31.600 | 17.600 | 14.000 |
| 17 | ES6 | -19.800 | -40.117 | 20.997 | 1171.00 | 187.000 | 39.000 | 8.717 | 182.606 | 31.700 | 17.200 | 14.500 |
| 18 | ES7 | -19.617 | -40.150 | 32.996 | 1192.00 | 190.000 | 35.000 | 9.133 | 181.262 | 31.700 | 16.900 | 14.800 |
| 19 | ES8 | -19.877 | -40.161 | 45.997 | 1154.00 | 188.000 | 39.000 | 8.708 | 179.951 | 31.600 | 17.300 | 14.300 |
| 20 | ES9 | -20.416 | -41.010 | 1255.930 | 1408.98 | 219.998 | 37.999 | 10.833 | 180.559 | 25.400 | 8.500 | 16.900 |
| 21 | ES10 | -19.835 | -40.418 | 292.977 | 1202.00 | 198.000 | 36.000 | 9.558 | 174.166 | 30.700 | 15.800 | 14.900 |
| 22 | ES11 | -19.870 | -40.870 | 216.016 | 1171.01 | 205.000 | 27.000 | 11.000 | 180.697 | 32.400 | 15.500 | 16.900 |
| 23 | ES12 | -19.468 | -40.119 | 22.002 | 1196.00 | 189.000 | 35.000 | 9.158 | 177.138 | 31.600 | 16.900 | 14.700 |
| 24 | ES13 | -19.628 | -39.860 | 5.002 | 1214.00 | 183.000 | 45.000 | 8.333 | 180.362 | 31.200 | 17.400 | 13.800 |
| 25 | ES14 | -20.033 | -40.683 | 717.037 | 1284.01 | 207.001 | 37.001 | 10.250 | 174.419 | 28.600 | 12.700 | 15.900 |
| 26 | ES15 | -20.062 | -40.744 | 808.997 | 1304.00 | 210.000 | 37.000 | 10.483 | 176.652 | 28.200 | 11.900 | 16.300 |
| 27 | ES16 | -20.042 | -40.702 | 738.011 | 1290.00 | 208.000 | 37.000 | 10.350 | 176.607 | 28.500 | 12.500 | 16.000 |
| 28 | ES17 | -19.905 | -40.694 | 613.935 | 1266.98 | 207.999 | 35.000 | 10.367 | 174.745 | 29.300 | 13.300 | 16.000 |
| 29 | ES18 | -19.973 | -40.530 | 718.928 | 1293.98 | 204.999 | 40.999 | 9.825 | 171.960 | 28.300 | 13.000 | 15.300 |
| 30 | ES19 | -19.889 | -40.546 | 839.934 | 1321.99 | 206.999 | 40.000 | 10.025 | 171.364 | 27.700 | 12.100 | 15.600 |
| 31 | ES20 | -19.925 | -40.623 | 633.916 | 1271.98 | 206.999 | 36.000 | 10.233 | 173.741 | 29.101 | 13.301 | 15.800 |
| 32 | ES21 | -19.938 | -40.594 | 767.963 | 1301.99 | 206.999 | 39.000 | 10.092 | 172.971 | 28.200 | 12.500 | 15.700 |
| 33 | ES22 | -20.465 | -41.002 | 1216.040 | 1399.01 | 219.000 | 38.001 | 10.825 | 181.713 | 25.600 | 8.700 | 16.900 |
| 34 | RJ1 | -21.882 | -41.832 | 1213.790 | 1478.95 | 237.995 | 38.997 | 10.217 | 201.923 | 24.301 | 7.901 | 16.400 |
| 35 | RJ2 | -22.425 | -42.038 | 74.014 | 1155.01 | 184.002 | 35.000 | 8.783 | 200.356 | 29.900 | 15.000 | 14.900 |
| 36 | RJ3 | -22.317 | -42.333 | 1037.980 | 1438.00 | 247.999 | 29.999 | 9.950 | 213.082 | 25.800 | 9.000 | 16.800 |
| 37 | RJ4 | -22.398 | -43.157 | 816.014 | 1777.99 | 300.000 | 43.999 | 9.692 | 224.170 | 27.000 | 10.300 | 16.700 |
| 38 | RJ5 | -21.736 | -41.036 | 3.001 | 1026.00 | 148.000 | 27.000 | 8.667 | 191.433 | 30.200 | 15.400 | 14.800 |
| 39 | RJ6 | -22.786 | -42.124 | 21.998 | 944.00 | 132.000 | 39.000 | 7.125 | 185.106 | 29.200 | 16.800 | 12.400 |
| 40 | RJ7 | -22.549 | -42.277 | 34.005 | 1122.00 | 174.001 | 33.000 | 8.558 | 201.420 | 30.200 | 15.600 | 14.600 |
| 41 | SE1 | -11.300 | -37.550 | 84.996 | 1315.02 | 218.005 | 44.001 | 8.875 | 155.933 | 31.600 | 18.500 | 13.100 |
| 42 | SE2 | -11.200 | -37.233 | 21.994 | 1487.98 | 268.998 | 50.999 | 7.050 | 120.175 | 30.600 | 20.300 | 10.300 |
